# Clinical and molecular markers in retinal detachment—From hyperreflective points to stem cells and inflammation

**DOI:** 10.1371/journal.pone.0217548

**Published:** 2019-06-11

**Authors:** Natasha Josifovska, Xhevat Lumi, Mária Szatmari-Tóth, Endre Kristóf, Greg Russell, Richárd Nagymihály, Natalia Anisimova, Boris Malyugin, Miriam Kolko, Domagoj Ivastinović, Goran Petrovski

**Affiliations:** 1 Center for Eye Research, Department of Ophthalmology, Oslo University Hospital and University of Oslo, Oslo, Norway; 2 Eye Hospital, University Medical Centre, Ljubljana, Slovenia; 3 Department of Biochemistry and Molecular Biology and MTA-DE Stem cell, Apoptosis and Genomics Research Group, University of Debrecen, Debrecen, Hungary; 4 Department of Ophthalmology, Faculty of Medicine, University of Szeged, Szeged, Hungary; 5 S. Fyodorov Eye Microsurgery State Institution, Moscow, Russian Federation; 6 Department of Drug Design and Pharmacology, University of Copenhagen and Department of Ophthalmology, Copenhagen University Hospital, Rigshospitalet-Glostrup, Copenhagen, Denmark; 7 Department of Ophthalmology, University of Graz, Graz, Austria; University of Florida, UNITED STATES

## Abstract

**Purpose:**

Retinal detachment (RD) is one of the most frequently diagnosed ophthalmologic conditions requiring prompt surgical intervention. Combination of proper surgical technique and new diagnostic markers, both clinical and molecular, can help improve the diagnosis and prognosis of RD treatment.

**Methods:**

12 patients with rhegmatogenous RD (rRD) were included into the study after obtaining patient consent and Regional Ethical Approval (average age: 58.1 ± 17.4 years). OCT was performed before and after 23G vitrectomy for RD. Pure subretinal fluid (SRF) was collected during surgery and analyzed by protein array profiling on a panel of 105 inflammatory cytokines (Human XL Cytokine Array), while the effect of SRF upon human macrophages-driven phagocytosis of apoptotic retinal pigment epithelial (RPE) cells *ex vivo* was quantified by flow cytometry. Immunohistochemistry (IHC) of retinectomized tissue due to PVR caused by RD was performed to determine presence of markers for microglial cells (CD34), macrophages and activated microglia (CD68), regulator of the immune response to infection (NFkB), progenitor and stem cell marker (Sox2), pluripotency marker (Oct4) and intermediate filament markers (GFAP and Nestin).

**Results:**

OCT of fresh RD patients contained pre-operatively hyper reflective points (HRPs) at the detached neuroretina border and proximal to the RPE layer—their size and number decreased following successful reattachment surgery. IHC of the retinectomized tissue from detached retina due to severe PVR showed presence of cell conglomerates at the detached neuroretina border which were positive for CD68, NFkB, Sox2 and GFAP, less positive for CD47 and Nestin and negative for Oct4 and CD34. The SRF contained at least 37 cytokines with higher, and 4 cytokine with lower concentration compared to that in vitreous from non-RD pathology; when used as conditional medium to human macrophages *ex vivo*, the SRF doubled their capacity for engulfing dying RPEs.

**Conclusions:**

Fresh RD can be hallmarked by presence of HRPs at the detached neuroretina border on OCT; the HRPs decrease in size and number after successful reattachment surgery, and likely resemble the macrophage conglomerates seen by IHC. The neuroretina in RD contains progenitor/stem-like cells and signs of inflammatory reaction, while the SRF contains inflammatory cytokines and other factors which increase the ability of professional phagocytes to engulf dying RPE, or for that matter, other dying cells in the retina.

## Introduction

Retinal detachment (RD) is one of the most serious and damaging disorders of the retina and vision, requiring prompt surgical action. The incidence of RD ranges from 1:10000–15000 per year, most patients being 50 years of age or older [1, 2]. Delayed or improper surgical treatment of patients in the acute phase of the disease can cause severe to permanent visual impairment. Beside the clinical signs seen by ophthalmoscopy, ultrasound and alternatively, optical coherence tomography (OCT) can be used to confirm the diagnosis.

OCT is a light-based imaging modality that can be used in biological systems to study tissues *in vivo* with near-histological, ultrahigh resolution [3–5]. Hyperreflective points (HRPs) have been detected by OCT and studied in relation to diseases like retinitis pigmentosa [6], macular holes [7], diabetic macular edema [4], age-related macular degeneration [8], adenovirus keratoconjunctivitis [9] or uveitis [10]. It has also been shown that such HRPs are aggregates of activated microglia cells [11]. Their presence, number and location serve as a prognostic factor in many of these diseases.

We hereby present a study in which OCT scans of eyes with fresh rhegmatogenous RD (rRD) were performed before and after RD surgery to observe for presence or change of the number of HRPs in the neuroretina and near the border with the retinal pigment epithelium (RPE), from which the neuroretina got detached. Correlation with cellular aggregates found by immunohistochemistry on retinectomized tissue due to proliferative vitreoretinopathy (PVR) caused by RD was performed to determine presence of markers for microglia (CD34), macrophages and activated microglia (CD68), regulator of the immune response to infection (NFkB), progenitor and stem cell marker (Sox2), pluripotency marker (Oct4) and intermediate filament markers (GFAP and Nestin).

Furthermore, the subretinal fluid (SRF) found between the neuroretina and the underlying RPE layer, which is secreted by the RPE cells, was studied since its composition is still not fully known. It is assumed that the SRF contains cytokines which play an important role in the RD, which is actually a sterile form of inflammation [12].

The present study aimed to find a reliable clinical marker which can be a putative marker for RD as well as prognostic factor for surgical success or outcome, next to finding molecular markers such as presence of inflammatory cytokines in the SRF, and the effect of SRF upon dead cell clearance in the retina.

## Materials and methods

### Tissue collection and cultivation of cells

All tissue collection complied with the Guidelines of the Helsinki Declaration (1964) and was approved by the National Medical Ethics Committee of the Republic of Slovenia (Ref. No. 112/01/13). Twelve patients with rRD (7 females, 5 males), all having detached macula, were included in the study after written informed consent was obtained. Average age of the patients was 58.1 ± 17.4 years.

### OCT examination and HRP quantification

12 rRD patients underwent an OCT scan of the retina during the study (Nidek RS-3000 Advance). Two images were made from each eye before and after repair surgery for RD (23G pars plana vitrectomy) upon clear optical media appearance. The images were compared in the same level plane with special regard to the presence of HRP at the two time points.

Quantification of the HRPs was initially performed manually, and then by a less subjective interpretation. The original tiff files were segmented by adjustment of brightness at numerical 68 contrast at numerical 123 within the levels tool in Image J. The dynamic range threshold was adjusted to help isolate the cells of interest and subtract the background, then a “Contrast Limited Adaptive Histogram Equalization” (CLAHE) filter was used to normalize the contrast values. The cell shape and size were present as between 20–40 pixels in diameter and measured within the Region of Interest (ROI) selected equally for OCT images analyzed.

### Immunohistochemical (IHC) analysis

Paraffin embedded sections fixed in formalin (4%) from retinectomized tissue due to severe PVR caused by RD were analyzed by classical Hematoxylin & Eosin (H&E)- and immune-staining for presence of microglia cell marker (CD34) and viability/’don’t-eat-me’ signal marker (CD47). Presence of macrosialin—a heavily glycosylated transmembrane protein of 87-115kD, which is specifically expressed by tissue macrophages, Langerhans cells and at low levels by dendritic cells—the murine homologue of the human macrophage glycoprotein (CD68) was checked. Furthermore, (NFkB)-regulator of the immune response to infection, (Sox2)-progenitor and stem cell marker, (Oct4)-pluripotency marker and (GFAP and Nestin)-intermediate filament markers were checked. Incubation was carried out at room temperature for 60 minutes. The chromogen substrate was DAB with incubation lasting for 1–3 minutes at room temperature.

### SRF sample collection and measurement of inflammatory cytokines

Undiluted/pure SRF samples were obtained during vitrectomy for primary rRD using a 41G needle. Control samples/vitreous were obtained from uneventful vitrectomies (VC) for non-RD pathology (e.g. epiretinal fibrosis surgery). All samples were collected in sterile polypropylene tubes and stored at −20°C until analysis. Sample volumes ranged between 100–700 μL.

A semi-quantitative array (Proteome Profiler, Human XL Cytokine Array Kit, Bio-Techne, USA) was purchased to determine the cytokine profile of the SRF and non-RD vitreous body. Samples were thawed on ice and prepared according to the manufacturer’s guidelines. Briefly, 4 donors of SRF and 4 donors of control vitreous body were pooled (to reduce the inter-donor variability) and centrifuged at 2500 RPM for 5 minutes to sediment any debris. The membranes (arrays) with the antibodies were blocked, then the samples were applied on two separate arrays and incubated overnight at 4°C with agitation. The following day, after washing the membranes, a cocktail of detection antibodies was applied on the arrays, followed by an incubation in a streptavidin-HRP solution. For visualization, a chemiluminescent reagent was pipetted on the membranes and images were taken by an ImageQuant LAS 4000 mini device. The membranes were exposed to light for 10 minutes and images were recorded.

Dot blot analysis was carried out using ImageJ. Briefly, the background was subtracted from the image and the ‘raw integrated density’ of each individual dot (printed in duplicates) was determined (sum of the values of the pixels in the selection). Results were visualized in Microsoft Excel.

### *In vitro* analysis of the effect of SRF upon the clearance of dying RPE cells by human macrophages

Quantification of the phagocytic capacity of macrophages engulfing anoikic dying ARPE cells in presence or absence of SRF (50:50% conditional medium) was performed. Human monocytes were isolated from ‘buffy coats’ of healthy blood donors on Ficoll–Paque Plus (Amersham Bio-sciences, Uppsala, Sweden) gradient and magnetic separation using CD14 human microbeads (Miltenyi Biotec, Auburn, CA, USA). Human macrophages were obtained through a 5-day differentiation using 5 ng/ ml macrophage colony-stimulating factor (Peprotech EC, London, United Kingdom). Dying ARPE-19 cells were fed to macrophages 24 hrs after the induction of anoikis. Pre-treatment of macrophages with the SRF conditional medium was performed 48 hrs prior to the assay. The macrophages were stained with 7.5 μM CMTMR (Invitrogen) for16 hrs, washed twice in phosphate-buffered saline (PBS) before starting the assay. The cells being engulfed were stained with 12.5 μM CFDA for 16 hrs before the phagocytosis and washed twice in PBS before being added to the phagocytes. The ratio of phagocytes and cells to be engulfed was set at 1:5. The phagocytosis assay started when the cells to be engulfed were added to the appropriate phagocytes and kept together for 4 hrs. The assay was ended by trypsinizing the phagocytic cell mixture to remove bound but not engulfed dying cells, centrifuging, washing twice in PBS and fixing in 1% PBS-buffered paraformaldehyde (pH 7.4). The phagocytosis rate was determined by FACS analysis as percent phagocytic cells (CMTMR positive) that have engulfed dying cells (positive for both CMTMR and CFDA) as described by our group previously [13].

## Results

### Presence of HRP on OCT before and after surgery for rRD

**Fig 1:**
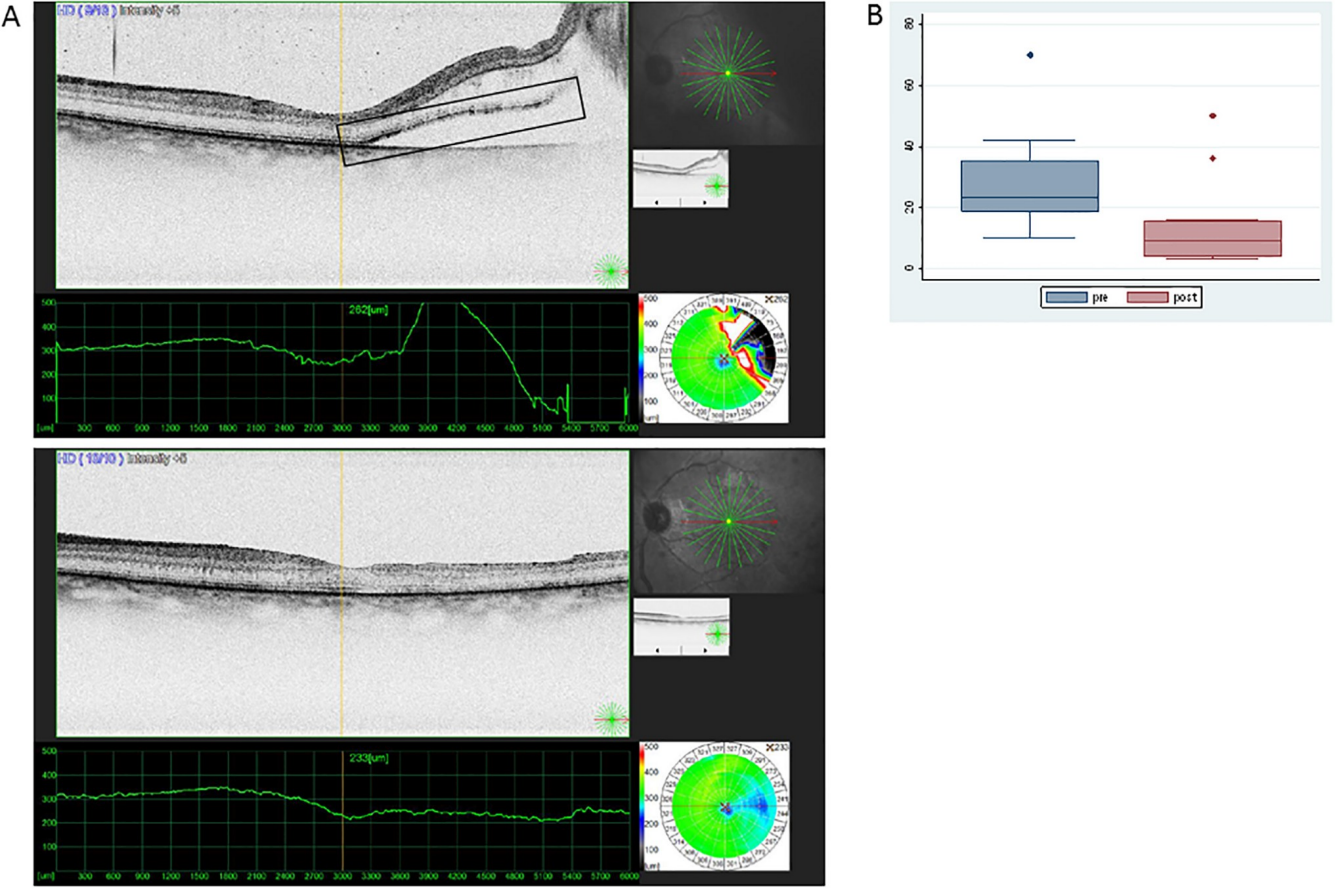
Presence of HR points in the retina before and after surgery for rRD. (A) HR points are shown in the square box. OCT image from the retina when detached (image above) and after reattachment surgery (image below) showing absence of subretinal fluid and diminishing presence of HR points in the neuroretina. (B) Medians and Interquartile range of number of HR points counted on OCT images during the pre- (RD) and post- (reattachment) state (outliers being shown as separate dots).

OCT scans were performed on all patients studied with RD: one before surgery, while the detachment was still fresh, and one after surgery, upon clear media appearance. HRPs were detected before surgery in the neuroretina, more abundantly in the outer retinal layers of the neuroretina, near the detached border from the RPE layer. The HRPs decreased in size and number upon successful reattachment following vitrectomy (Fig 1A and 1B).

**Fig 2:**
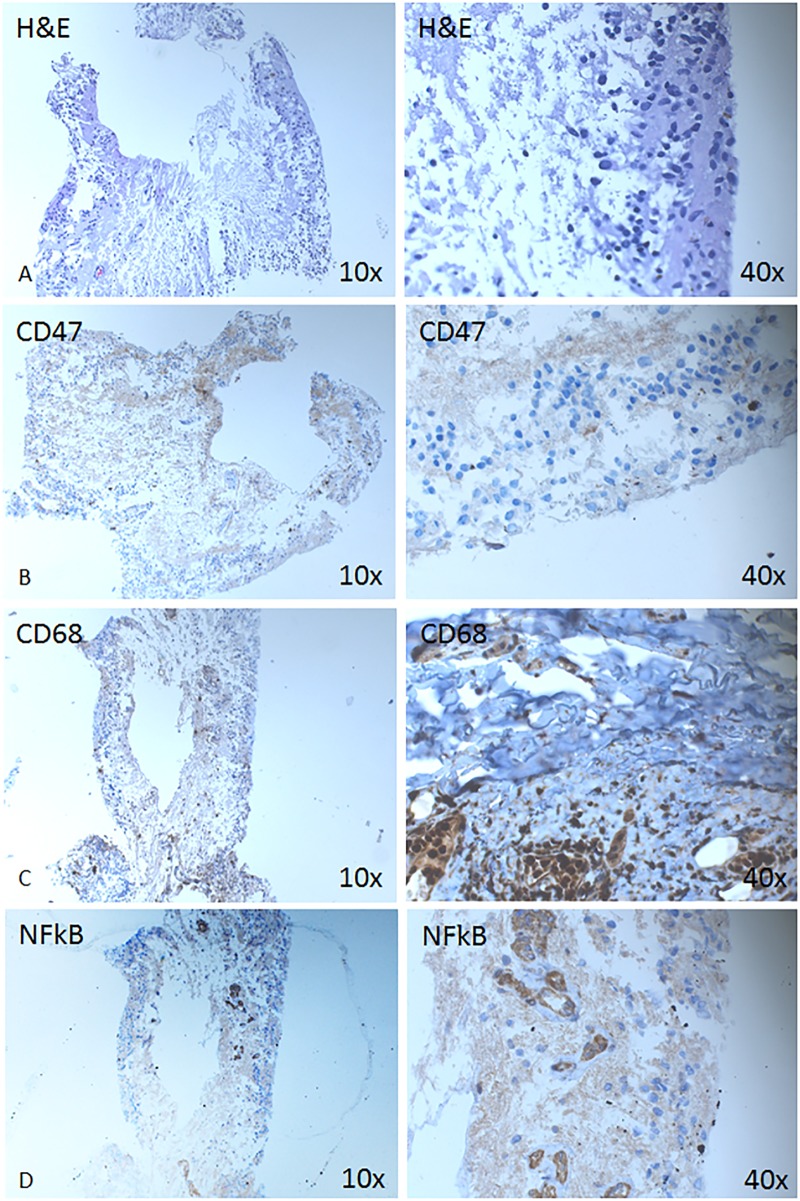
Expression of hematopoietic, macrophage and inflammatory pathway markers is shown on a retinectomized tissue from RD with PVR. Immunohistochemical analysis is shown against CD47 (B), CD68 (C) and NFkB (D) at magnification 10X (left column) and 40x (right column).

**Fig 3:**
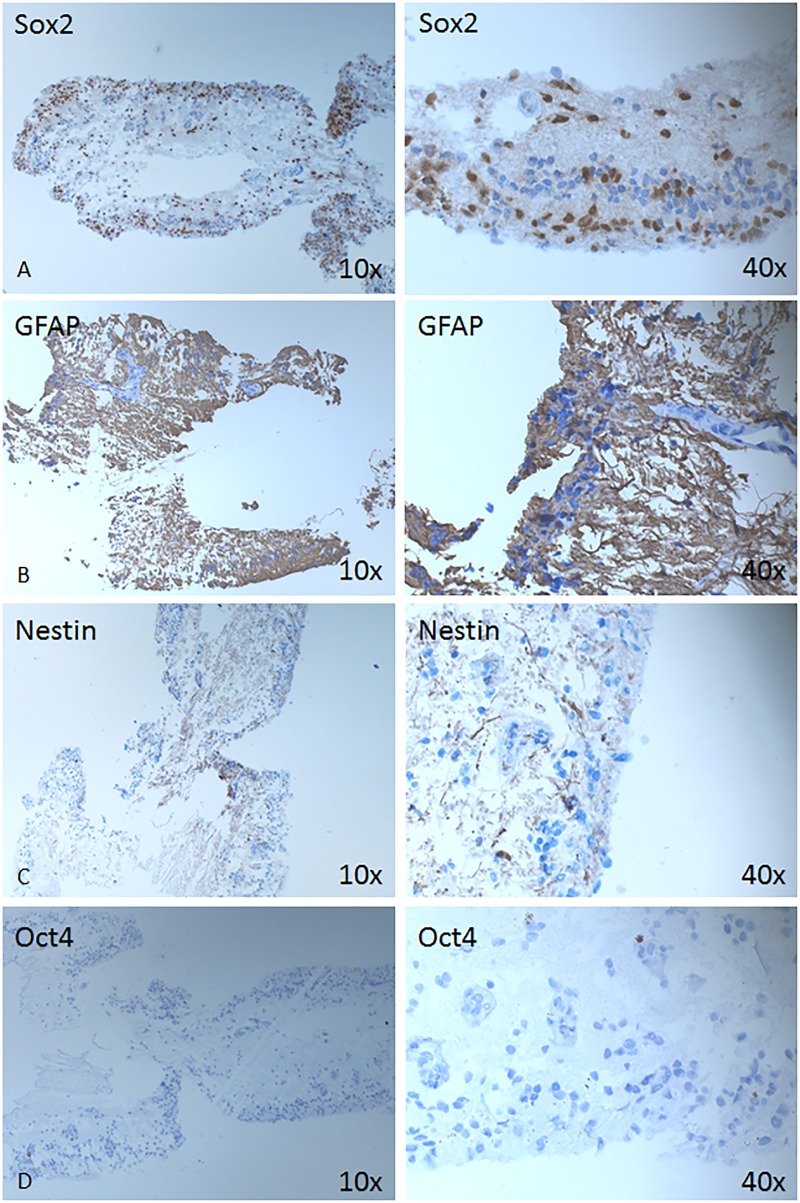
Expression of stem/progenitor cell-, neuro-glial markers. Immunohistochemical analysis is shown against Sox2 (A), GFAP (B), Nestin (C) and Oct4 (D) at magnification 10X (left column) and 40x (right column).

Immunohistochemical analysis was performed on detached neuroretina which was retinectomized due to presence of PVR, to see the structural changes and detect the expression of different markers in the tissue (Figs 2 and 3). The IHC low expression of CD47 positive cells (Fig 2B). Cellular aggregates positive for CD68 (marker for all types of macrophages, but likely M2 type of macrophages or alternatively activated ones) could be detected (Fig 2C). Furthermore, the detached neuroretina was positive for NFkB (Fig 2D), which plays a role in the expression of pro-inflammatory genes includiwng cytokines, chemokines, and adhesion molecules [14], as well as Sox 2—progenitor and stem cell marker (Fig 3A), and the intermediate filament marker GFAP (Fig 3B), while being somewhat less positive for Nestin (Fig 3C) and negative for Oct4 (Fig 3D).

### Semi-quantitative analysis of inflammatory cytokines in the SRF from RD

**Fig 4:**
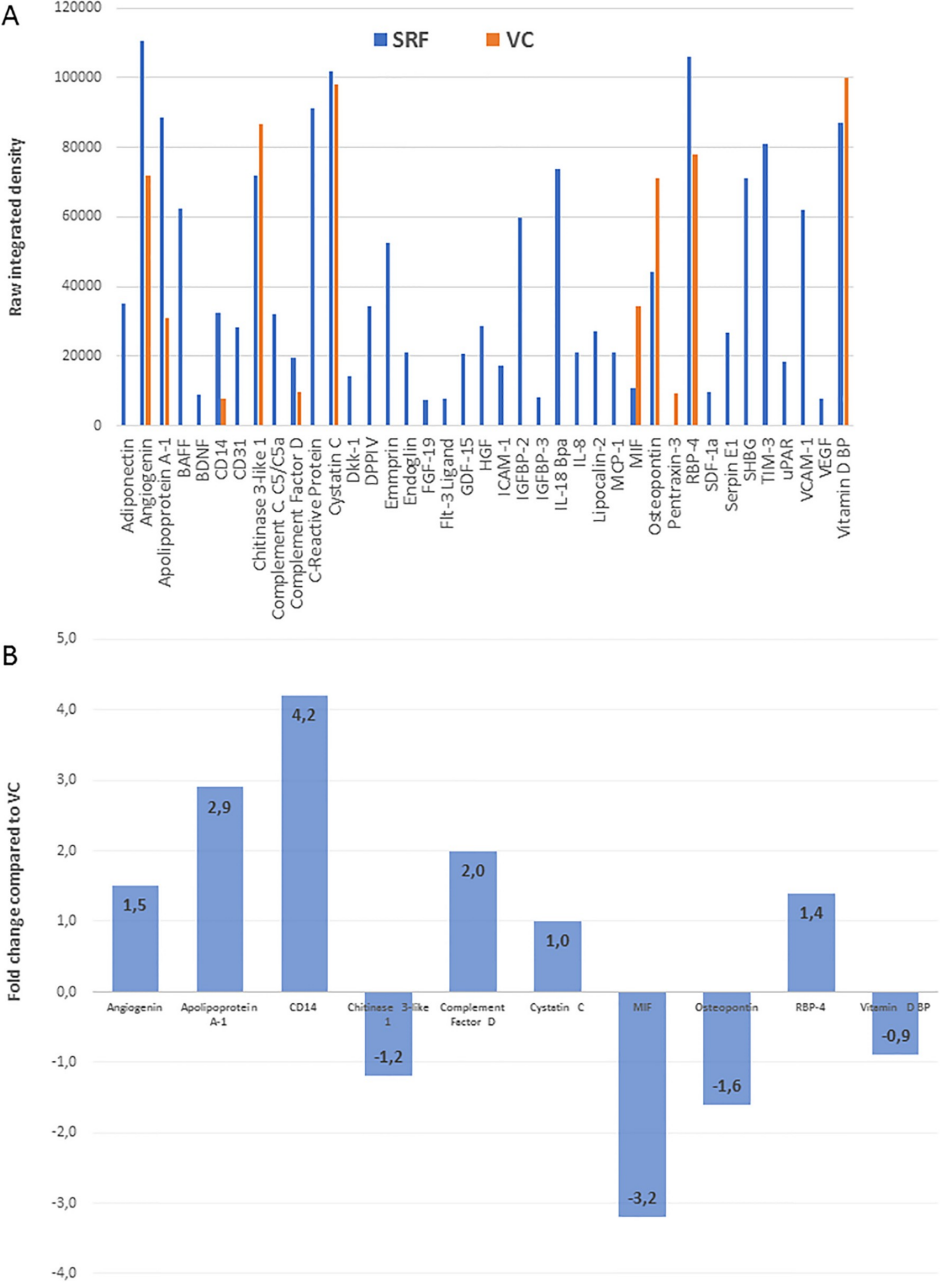
Semi-quantitative proteome profiler cytokine array. Individual cytokines detected in SRF (blue bars) versus VC (orange bars) are plotted against pixel density values (y-axis) (A). The difference in the cytokines detected in both SRF and VC are represented (B) as the fold change difference in SRF compared to VC.

38 cytokines were identified in the SRF samples: Adiponectin, Angiogenin, Apolipoprotein A-1, BAFF, BDNF, CD14, CD31, Chitinase 3-like 1, Complement Component C5/C5a, Complement Factor D, C-Reactive Protein, Cystatin C, Dkk-1, DPPIV, Emmprin, Endoglin, FGF-19, Flt-3 Ligand, GDF-15, HGF, ICAM-1, IGFBP-2, IGFBP-3, IL-18 Bpa, IL-8, Lipocalin-2, MCP-1, MIF, Osteopontin, RBP-4, SDF-1α, Serpin E1, SHBG, TIM-3, uPAR, VCAM-1, VEGF and Vitamin D BP. The VC contained 11 individual cytokines: Angiogenin, Apolipoprotein A-1, CD14, Chitinase 3-like 1, Complement Factor D, Cystatin C, MIF, Osteopontin, Pentraxin-3, RBP-4 and Vitamin D BP (Fig 4A).

Increased level of secretion of Angiogenin (1.5-fold), Apolipoprotein A-1 (2.9-fold), CD14 (4.2-fold), Complement Factor D (2.0-fold) and RBP-4 (1.4-fold) were detected in the SRF compared to the controls, while decreased levels were detected for Chitinase 3-like 1 (1.2-fold), MIF (3.0-fold), Osteopontin (1.6-fold) and Vitamin D BP (0.9-fold), accordingly. (Fig 4B) Cystatin C was detected in both SRF and control samples in equal quantities, while Pentraxin-3 was detected in the controls only and not the SRF. The remaining 27 cytokines were identified exclusively in the SRF and were not detectable in the vitreous controls.

### SRF enhances phagocytosis of dying RPE cells by macrophages

**Fig 5:**
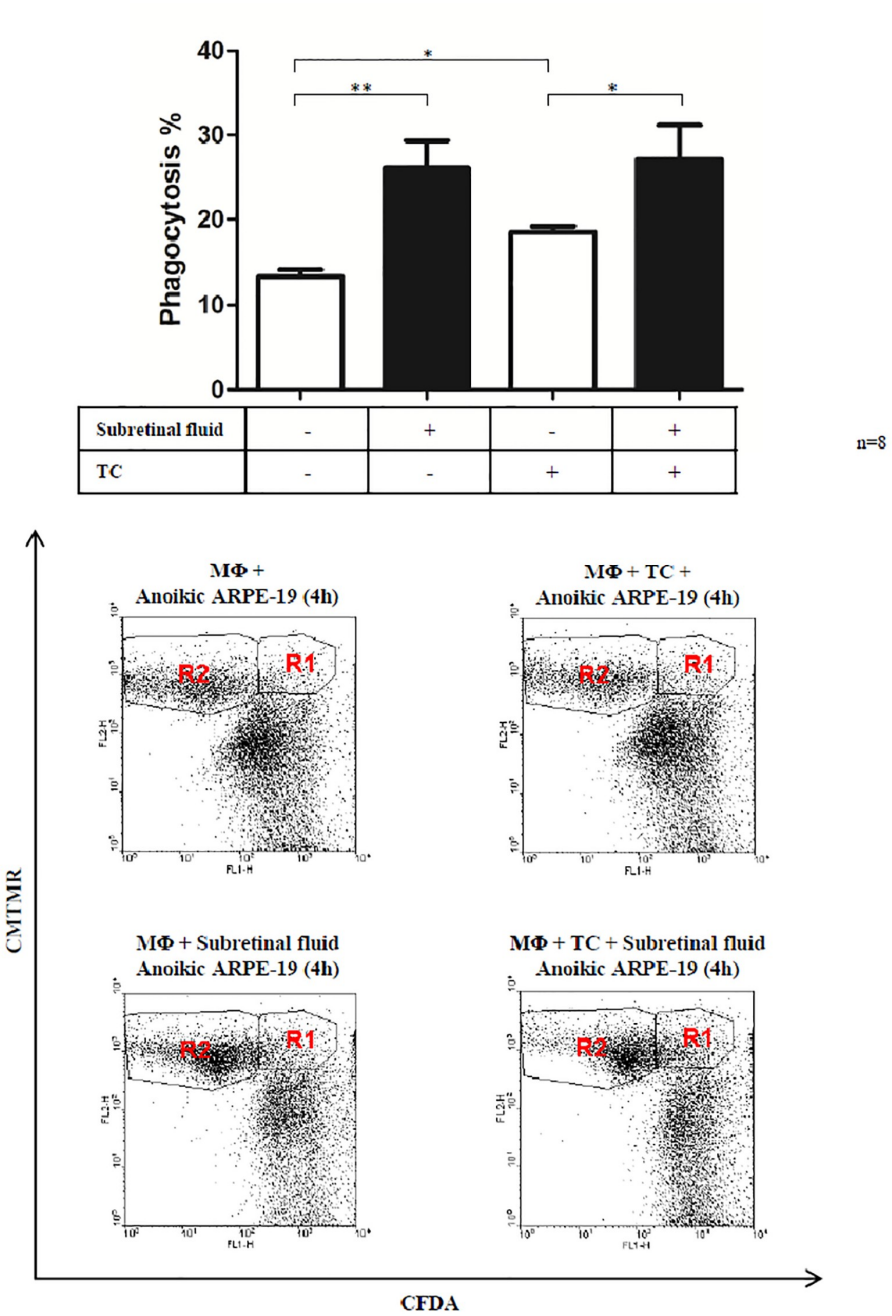
Phagocytosis study of macrophages (MΦ) engulfing dying ARPE-19 cells upon treatment with conditional medium containing 50% subretinal fluid, as well as triamcinolone (TC). The dot plots are representative images of the flow cytometry analysis of the engulfment process under the shown conditions (R1 represents the population of macrophages which have engulfed dying ARPE-19 cells, while R2 represents macrophages which have not engulfed dying cells). (*) represent statistical significance (p<0.05) of n = 8 samples.

The SRF/conditional medium enhanced significantly or two-fold the phagocytosis percent carried out by macrophages engulfing dying ARPE-19 cells (p<0.05). This phagocytosis enhancing effect of the SRF was higher than that caused by the known enhancer of the phagocytosis process (triamcinolone, TC) and showed no additive or cumulative effect upon TC+SRF treatment (Fig 5).

## Discussion

A RD is relatively common and severe disorder of the eye which involves cellular mechanisms such as inflammation and cell death in its pathogenesis. The inflammation is sterile without any presence of infection. The most common form of RD is rRD, which occurs as a result of a full-thickness retinal break. Vitreoretinal traction takes role in rRD by allowing accumulation of liquefied vitreous under the retina leading to its separation from the RPE. Accordingly, there are few precursors to such a type of RD, including liquefied vitreous, tractional forces and break through which fluid gains access to the subretinal space [15, 16].

The pathomechanism of RD can be divided into actions occurring during the acute phase of the detachment, and actions during the chronic phase, which is hallmarked by proliferative vitreoretinopathy (PVR). The earliest structural effects of RD can be seen on the outer segments (OS) of the photoreceptors and RPE cells. Between these two structures, there are no actual cellular junctions in the mature eye, but they are adherent through the numerous microvilli present on the apical surface of the RPE. Upon RD, these connections become damaged and a space between the two cellular layers is formed. The subretinal space is usually free of cellular content, but during RD, cells like neutrophils, monocytes and macrophages can migrate into the damaged area together with migrating RPE cells. The damaged OS of the photoreceptors also flow into the subretinal space, where they become phagocytosed by RPEs or other phagocytes present in the vicinity. Within 24 hours from RD, microglia-like cells have been shown to display signs of proliferation [17].

The precise nature of HRPs remains elusive [18]. HRPs have been previously studied in other eye disorders, and correlations to their microglial origin has been shown in some of them, but to the best of our knowledge, not in RD. It is known that microglial cells are important participants in the inflammatory response and, therefore, their presence can be presumed whenever inflammation is seen [19]. It has also been suggested that HRPs may be leucocytes or RPE cells, indicating retinal inflammation in itself. An alternative theory about the origin of HRPs is that they may represent small intraretinal proteins or lipid deposits, acting as a precursor of hard exudates or as an indication of lipoprotein extravasation, after the breakdown of the inner blood-retina barrier, usually present in patients with diabetic retinopathy (DR) and retinal vein occlusion. Furthermore, there are suggestions that HRPs are the result of neurodegenerative processes that occur in diabetes even before the development of clinical signs of DR [18]. Ideally, after surgical intervention, the neuroretina should return back to its original position, which is adherent to the RPE layer, accompanied by attenuation of the inflammatory cells. Decreased or remaining HRPs on OCT can therefore refer to remaining inflammation, thus provide a prognostic measure of the success of reattachment surgery. Nevertheless, recently, presence of residual SRF between the neuroretina and the RPE layer has been shown to be beneficial for the success of reattachment, probably due to presence of different factors, likely other than inflammatory factors, in the SRF, which may have beneficial effect in the cell survival and cell-to-cell reattachment.

Macrophages and microglial cells have several functions like scavenging, cytotoxic functions, antigen presentation, synaptic stripping, promotion of repair, extracellular signaling or phagocytosis. The microglial cells are normally active in the CNS and dormant in the retina, but during RD they, together with the monocyte-macrophage surveillance system can become activated and start to divide and/or migrate to the border of the neuroretina and RPE layer [20–22]. Several studies have shown that macrophages are recruited to the subretinal space through the Bruch’s membrane and the RPE monolayer during RD [23]. CD68, a cell surface marker for macrophages has been previously reported to be expressed by cultured RPE cells [24]. Macrophages have specific receptors such as lipopolysaccharide receptor CD14, phosphatidylserine receptors, the integrin receptor α_v_β_3_, and the scavenger receptors SRA1 and CD36 which take role in apoptotic cell removal by recognizing phosphatidylserine, oxidized lipids, and other unidentified phagocytic signals exposed by apoptotic cells. We could identify cell aggregates in the detached neuroretina retinectomized from RD with PVR to be positive for the macrophage marker CD68, and negative for microglial and M1 macrophage cell marker CD34, respectively. IHC could indeed reveal increased presence and aggregation of inflammatory/ phagocytic cells at the neuroretina / RPE border during RD, resembling very much the HRPs seen on OCT in a similar location. Furthermore, CD47, a member of the Ig cell adhesion molecule family, is expressed by virtually all cells in the body. Pharmacological activation of CD47 has been shown to accelerate resolution of subretinal and peritoneal inflammation, with implications for treatment of chronic inflammatory diseases and possibly other conditions [25–27].

NFkB an important regulator of the innate immune response, an inhibitor of some cell degradation/autophagic processes, and involved in aging is another marker that was expressed in the detached neuroretina. Its expression appeared to be limited, however, to the vascular endothelial cells, which may correlate with the finding of another study showing NFkB to play an important role in maintaining constitutive VEGF secretion in the RPE/choroid complex [28].

SOX2 is required at all major stages of retinal development for the proper formation of the mammalian eye and has an important role in maintaining neural progenitor/stem cell properties and in converting fibroblasts into pluripotent stem cells [29–31]. In the detached neuroretina, there was a high expression of this marker, indicating abundance of cells with potential to proliferate and differentiate into another cell type; meanwhile, no expression of the pluripotency marker Oct4 could be detected in the analyzed samples. It has been shown that silencing of the expression of Oct4 in mice Müller glial (MG) cells after retinal damage, and prevention of the silencing of this pluripotency-associated gene after injury, may possibly improve the transition from MG to retinal progenitor cells, leading to MG-mediated retina regeneration [32].

Upregulation of GFAP has been an indicator of stress in CNS astrocytes. The retina responds similarly to stress by upregulating this intermediate filament first in the MG cells. An increase in the GFAP in MG cells has indeed been shown after RD [33, 34] which could also be seen in our case in the detached neuroretina sample.

Nestin is another intermediate filament marker for neural progenitor cells that may play a part in the maintenance of the normal retina. Nestin positive cells in the adult human retina show morphological and geographical similarity to neurons, including retinal ganglion cells, other neurons, and MG cells [35, 36]. An upregulation of Nestin expression in the retinal microglia, macrophages, and MG cells has been shown before in adult mice undergoing pharmaceutical induction of retinal degeneration, implicating that Nestin may be used as an early-stage marker of acute retinal injury [37]. Following injury, however, microglial cells become activated and express Nestin, which appears to be the cells in some of the cells in our detached neuroretina.

The resident ocular cells react to damage stimuli by altering the chemokine levels in which an important role take the RPE cells following an appropriate stimulus. Contact with the vitreous or monocytes, stimulation by pro-inflammatory cytokines, and mechanical injury have all been shown to be a trigger for the production and secretion of chemokines by RPEs [12]. During RD, SRF fills out the gap between the detached neuroretina and the RPE layer, becoming a ‘flooded’ area for inflammatory cytokines and chemokines. These cytokines have several functions like regulation of different pathways including apoptosis and proliferative changes in the retina, leading to PVR. The definitive source of such factors is still not clear, but most likely a major source for their production are the monocytes and retinal glial cells like microglia or Müller cells that could have invaded the subretinal space [38]. Although the blood-retina barrier gets damaged during RD, the level of the plasma cytokines does not seem to reach high enough concentration to significantly contribute to the cytokine milieu found in the subretinal space. The levels of 38 cytokines and other growth factors (determined by us and other groups) appear to increase in the SRF, and contribute to the triggering and enhancement of the inflammatory and immune responses seen during RD [39–43] compared to the level of such cytokines in the vitreous of normal eyes. IL-6 plays an important role in inflammation and immune responses, chemokine induction and recruitment of immune cells like leukocytes as well as photoreceptor protecting function [39, 44, 45], which in the semi-quantitative protein array analysis of SRF could not be detected, but appeared enhanced in the more sensitive detection analysis of SRF by ELISA (S1 Fig). Similarly, IL-8 has a chemoattractant effect on neutrophils and RPE cells, and is mostly produced by RPE cells [46].

The results of the IHC have been corroborated by profiling the secretion of the most important inflammatory cytokines. Due to the RD, the usually cell-free subretinal spaced gets infiltrated by neutrophils and monocytes/ macrophages, joined by cellular debris and photoreceptor OS [17]. As a result of the breakup of tissue integrity and the pro-inflammatory cytokines secreted by the infiltrating monocytes/ macrophages, activation of the resident immune cells and RPEs occurs [23].

Macrophages have been shown to infiltrate the subretinal space through the RPE and through the Bruch`s membrane [12], which can be evidenced also by SRF-contained cytokines which are otherwise expressed in macrophages (CD14, CD31, ICAM-1, Osteopontin, MIF), and factors secreted by macrophages as a result of stimuli (Chitinase 3-like 1, IL-8, MCP-1). It is possible, that these factors enhance macrophages functions, such as phagocytosis (e.g. Complement Component C5/C5a, C- reactive protein, IL-8) [20], and assist the transmigration of more immune cells (e.g. CD31, Complement Component C5/C5a, ICAM-1, Osteopontin, VCAM-1). The exact source of the pro-inflammatory cytokines is unknown, however, our results suggest that macrophages play a major role in the process and these cells have been identified on our IHC sections. RD and the self- aggregating, pro- inflammatory process ultimately leads to proliferative vitreoretinopathy (PVR) formation, and as such, the presence of pro-inflammatory, fibrotic factors were found in the SRF (Chitinase 3-like 1, DPPIV, FGF-19, Serpin E1 [PAI-1], uPAR) along with other potent inducers of angiogenesis (Angiogenin, CD31, Endoglin, HGF, IL-8, SDF-1a, VEGF). To elaborate, whether inhibition of macrophage infiltration or function could dampen over-activation and prevent PVR needs to be further investigated with more exact, quantitative methods and a larger sample size.

Upregulation of the inflammatory mediators’ expression in the vitreous or SRF from patients with vitreoretinal disorders might have a negative effect upon the inflammation response and induce the proliferation and differentiation of RPE cells [47]. In our case, robust presence of stem/progenitor-like cells which were positive for Sox2 could be observed in the neuroretina from RD. Whether these cells are the source for the inflammatory or phagocytic cells or the consequence of the released inflammatory factors in the SRF by macrophages or other phagocytic cell types remains to be further studied. Besides cytokines and growth factors, the SRF contains some yet unknown factors which have enhancing effect upon phagocytic activity of macrophages and other cells of the immune system. This is clearly shown by the effect of our SRF conditional medium, which could enhance significantly the capacity of macrophages to engulf dying RPE cells *ex vivo*. In RD, the main function of the macrophages would be to phagocytose dying cells and help RPE cells in the clearance and survival/death processes. The role of specific and non- specific macrophages has been described in detail before [13, 20]. In addition, some of the factors detected and upregulated in SRF are angiogenic promoters and play roles in wound healing and tissue remodeling.

A limitation of the present and similar studies is that, to the best of our knowledge, no animal model exists for induction of RD rhegmatogenously, but rather mechanically, without presence of comparable ‘sterile’ inflammation as seen in humans *in vivo*. Furthermore, the use of semi-quantitative methods to study the proteome profile certainly needs validation by more sensitive methods like ELISA.

Overall, the present study shows for the first time the presence of HRP in the detached neuroretina layer which is put in correlation with the success of reattachment post-operatively. This is a relatively easy, simple, repeatable, non-invasive method for determining changes during RD and the success of surgery in the follow-up period. Presence of inflammatory and growth factors in the SRF as well as cellular reaction in the retina may serve as potential targets for drug therapy aimed at decreasing inflammation and cell death seen during RD.

## Supporting information

S1 Fig Concentration of IL-6 and IL-8 in the subretinal fluid from rRD determined by ELISA. Data shown are mean + S.D. from ten independent patient samples of SRF.
(TIF)
