# Supplementary figures and images for: Clinical and molecular markers in retinal detachment—From hyperreflective points to stem cells and inflammation
Source: PLoS One. 2019 Jun 11;14(6):e0217548. doi: 10.1371/journal.pone.0217548 (PMC6559703; doi:10.1371/journal.pone.0217548)

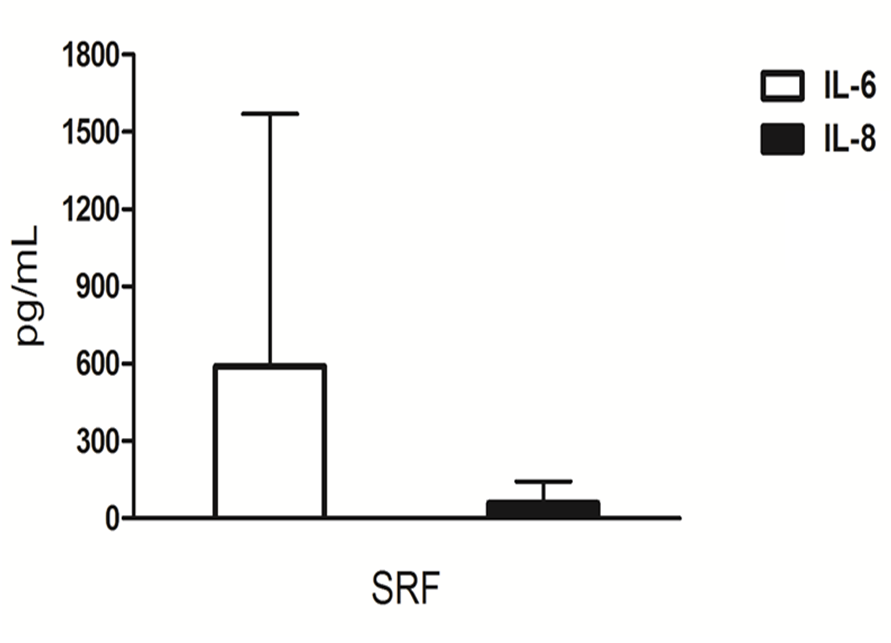

Supplement: S1 Fig — Data shown are mean + S.D. from ten independent patient samples of SRF. (TIF) [file pone.0217548.s001.tif]
